# Green revolution breeding favored water conservation, but weakened water use sensitivity to rising vapor pressure deficit in US spring wheat

**DOI:** 10.1093/plphys/kiag500

**Published:** 2026-07-14

**Authors:** Tina Koehler, Qiansu Ding, Emma Ossola, James Anderson, Andrea Carminati, Walid Sadok

**Affiliations:** Root-Soil Interaction, TUM School of Life Sciences, Technical University of Munich, Emil-Ramann-Str. 4/II, 85354 Freising, Germany; Department of Agronomy and Plant Genetics, University of Minnesota, Twin Cities, St. Paul, MN 55108-6026, United States; Physics of Soils and Terrestrial Ecosystems, Department of Environmental Systems Science, ETH Zurich, Universitätstrasse 16, 8092 Zürich, Switzerland; Department of Agronomy and Plant Genetics, University of Minnesota, Twin Cities, St. Paul, MN 55108-6026, United States; Physics of Soils and Terrestrial Ecosystems, Department of Environmental Systems Science, ETH Zurich, Universitätstrasse 16, 8092 Zürich, Switzerland; Department of Agronomy and Plant Genetics, University of Minnesota, Twin Cities, St. Paul, MN 55108-6026, United States

## Abstract

The global rise in vapor pressure deficit (VPD) threatens crop production. Plants mitigate high VPD by regulating their transpiration rate (TR). Yield is related to TR, but whether genetic yield gains reflect shifts in TR-VPD responses remains debated. Over the last century, US spring wheat yields have tripled in the Midwest. We aimed to determine (i) whether these gains relate to changes in water-use patterns under rising VPD and (ii) to identify underlying traits. We examined whole-plant TR responses to rising VPD under nonlimiting soil moisture in 14 wheat cultivars released over 105 years, representing a gradient in yield potential. Yield was quantified in a common trial, and morphological and hydraulic properties shaping TR-VPD responses were characterized under controlled conditions. Yield gains temporarily coincided with shifts in water-use patterns under rising VPD. Breeding has maintained a restricted water loss under high VPD in all cultivars. However, the tripling of yield potential around the Green Revolution coincided with the linearization of the TR-VPD response and a reduction in evaporative surface area in modern cultivars. This suggests a more effective water use rather than a reduced total water loss. Daily water-use regulation was associated with traits controlling water demand and supply. TR was restricted at lower VPD (VPD_BP_), but less effectively (higher slope after VPD_BP_, more linear TR-VPD response) in plants with a high water demand at low VPD, relative to their water channeling ability. These findings highlight opportunities to improve yields by fine-tuning hydraulic traits.

## Introduction

Climate change has led to a rise in vapor pressure deficit (VPD), which is likely to continue rising in the future (Yuan et al. 2019, 2025). A high VPD is the consequence of global warming combined with a comparatively lower increase in relative humidity (RH) (Ficklin and Novick 2017). The VPD, defined as the difference between saturated and actual vapor pressure, is an indicator of the “desiccating strength” of the atmosphere (Novick et al. 2024). An elevated leaf-level VPD (ie the difference between the quasi-saturated water vapor pressure inside the leaf at leaf temperature and the atmospheric water vapor pressure) drives high rates of transpiration from plants. For plants to limit their water loss, preserve their hydraulic integrity, and to maintain internal turgor pressures high enough to facilitate cell division and growth, stomata tend to close at high VPD (Grossiord et al. 2020). Stomatal closure, however, limits carbon assimilation (Sadok et al. 2019; Baker et al. 2023) and increases the risk of overheating (Gauthey et al. 2024). These and other acclimation-related VPD effects as discussed in detail in López et al. (2021) can lead to yield penalties under elevated VPD. This has been recognized as one of the key challenges for future agriculture (Lobell et al. 2014; Zhang et al. 2017; Novick et al. 2024) with yield losses being expected to further increase if mitigation strategies are not implemented (Kimm et al. 2020; Sun et al. 2023; Novick et al. 2024).

Genetic variability for the water use response to increasing VPD has been reported for major crops, revealing the existence of water spending and water-saving phenotypes (see review of Sinclair et al. 2017; Monnens and Sadok 2020). A dynamic stomata response that limits the increase in water loss (transpiration rate, TR) with rising VPD (ie water-saving phenotype) was suggested to confer increased resilience to agroecosystems under elevated VPD and terminal drought conditions with the benefit of enhanced soil water conservation that could be used during the critical seed-fill phase (Sinclair et al. 2005, 2010, 2017; Vadez et al. 2014; Messina et al. 2015; Sadok et al. 2019). However, the effect of a certain water use strategy on yield is highly context-specific (Hammer et al. 2006; Tardieu 2012; Monnens and Sadok 2020). For instance, in wheat grown in the Mediterranean environment of Tunisia, simulating a water-saving wheat phenotype expressing a restricted TR above a VPD threshold/breakpoint (VPD_BP_) of 2 kPa suggested yield gains in the dry, central parts of the country, but yield penalties in the north where precipitation is relatively higher, even if both regions qualify as water-limited (Sadok et al. 2019). Likewise, Schoppach and Sadok (2012) found that superior yields under drought could be achieved through both water-spending and water-saving phenotypes in Australian bread wheat. The water spending phenotype seems to be better suited to environments exposed to cyclic drought whereas the water-saving phenotype is more advantageous under terminal (progressive) drought conditions, where sustained water conservation is necessary to realize yield benefits (Izanloo et al. 2008; Schoppach and Sadok 2012). Thus, evaluating the effect of specific water use phenotypes on yield must carefully consider the water availability regime of the target environment.

Linking water use strategies to yield performance is a challenging effort, because grain yield is a highly complex and integrative trait. Mechanistically relating such strategies to agronomic yield performance often requires modeling and multiple location-years to generate the necessary environmental gradients needed to establish these links. An alternative experimental approach is to leverage “vintage trials” to examine the association between variation in candidate traits and genetic gains in yield achieved over decades of breeding (Ding et al. 2025). This approach consists of examining yield and target traits on widely adopted, locally relevant varieties released over a given time window. This method, pioneered by Fischer et al. (1998), offers the unique advantage of detecting signatures of “cryptic” or indirect selection of breeders of physiological traits that likely contributed to yield increase (Baca Cabrera et al. 2025; Ding et al. 2025). Historical yield increases, however, have not been linear, particularly for grain crops such as wheat. This is because the rate of genetic improvement is contingent on the rate of development and deployment of technological innovation which is not a linear process. For instance, the introduction of semi-dwarfing rht (reduced height) genes has been shown to be a contributing factor to the substantial increases in wheat yields starting from the mid-1960s (Fig. S1; USDA NASS), driving the so-called green revolution (Gill et al. 2026).

The first goal of this study was to test the hypothesis that changes in the TR sensitivity to rising VPD and related candidate hydraulic and morphological traits controlling water acquisition and loss during elevated atmospheric demand could be linked to genetic gains in yield. To this end, we combined a 2-year field experiment examining genetic gains in agronomic grain yield among a group of 14 widely used spring wheat varieties released in Minnesota between 1915 and 2022 with characterizing the TR sensitivity to increasing VPD and relate traits of the same genotypes under controlled conditions. Breeding for yield may have unintentionally altered plant water use strategies under rising VPD (Schoppach et al. 2017), given their potential contribution to yield formation. Because these cultivars were developed for the temperature environments of the Upper Midwest, we hypothesized that older varieties would exhibit a more linear TR response to increasing VPD, whereas more recently released varieties may show a water-saving phenotype (ie restricted TR response to rising VPD). This expectation is based on evidence showing a “weakening” of TR sensitivity to VPD leading more risk-taking (less conservative) behavior in Australian cultivars (Schoppach et al. 2017).

A first set of traits considered in this investigation consists of parameters characterizing the relation between TR and VPD. Namely the initial slope of the TR-VPD response (Slope1), the VPD upon which the slope in TR changes with rising VPD (VPD_BP_), the slope after the VPD_BP_ (Slope2), the difference in slope before and after the VPD_BP_ (Slope_diff._), total water loss over the course of the day at the whole plant level (ie without normalizing by leaf area, TWL) and as normalized by leaf area (ie cumulative TR per day, TR_tot_), nocturnal water loss (TR_night_), and its ratio to daytime TR (TR_night_ TR_day_^−1^), and transpiration efficiency (TE). A second set of developmental and anatomical traits consisted of leaf area (LA), specific leaf area (SLA), leaf blade dry mass, root biomass, root to shoot ratio, plant hydraulic conductance (K_plant_), stomatal density (SD).

Additionally, here we aimed to investigate how plant hydraulic traits contribute to shaping plant water use responses to rising VPD. Plant hydraulic traits are crucial for determining plant physiological functions under drought, as they shape photosynthesis, growth and overall plant performance, especially when water is limiting (Torres-Ruiz et al. 2024; Cardoso et al. 2025). The involvement of soil-plant hydraulics in shaping stomatal response to rising VPD is debated (Scoffoni et al. 2023; Bourbia and Brodribb 2024; Koehler et al. 2024). Stomatal closure at high VPD, inducing limitations in TR, is often viewed to be a response of the guard cells to a decline in local leaf water status (ie water potential) via a drop in turgor pressure vs. via phytohormone-mediated (particularly abscisic acid, ABA) changes in guard cell osmotic potential (Buckley 2005; McAdam and Brodribb 2016; Merilo et al. 2018). Because phytohormone synthesis under increasing VPD is itself driven by a decrease in leaf water potential (McAdam and Brodribb 2016) and by osmotically regulated turgor dynamics (Passioura and Fry 1992; Baca Cabrera et al. 2020), traits that govern the trajectory of leaf water potential with increasing TR during rising VPD (ie hydraulic traits) are critical in both scenarios.

**Figure 1:**
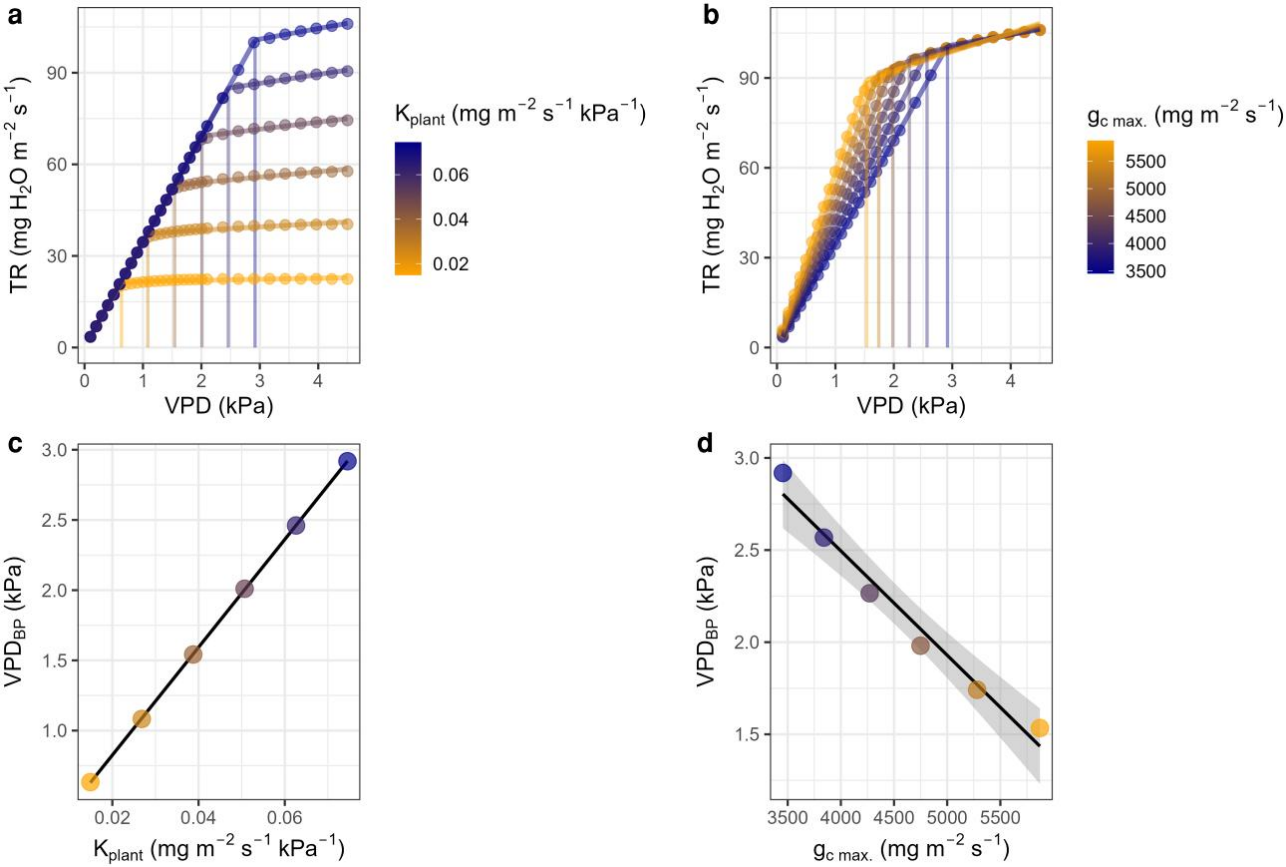
a, b) Relationship between the transpiration rate (TR) and vapor pressure deficit (VPD) as simulated by the soil-plant hydraulic model after Carminati and Javaux (2020). a) For a range of plant hydraulic conductances (K_plant_, 0.015 to 0.074 mg m^−2^ s^−1^ kPa^−1^) at a given maximum canopy conductance (3,454 mg m^−2^ s^−1^). b) For a range of maximum canopy conductances (g_c max._, 3,454 to 5,872 mg m^−2^ s^−1^) at a given plant hydraulic conductance (0.074 mg m^−2^ s^−1^ kPa^−1^). c, d) Summary of the dependence of the VPD breakpoint (VPD_BP_ = x-intercept of the vertical lines in (a) and (b), ie the VPD upon which water conservation due to stomatal closure is larger than water loss as driven by increasing evaporative demand) on c) K_plant_ and d) g_c max._, based on (a) and (b).

Our hypotheses are based on soil-plant hydraulic principles (Sperry and Love 2015; Carminati and Javaux 2020). Plants with a higher water-channeling ability (plant hydraulic conductance ie plant water supply) would experience less steep gradients in leaf water potential as TR increases. We expect such plants to show a linear TR response that extends to higher VPD levels (Fig. 1a, Sinclair et al. 2008; Choudhary et al. 2014). In contrast, plants losing more water per unit of leaf area and time at a given VPD (plants with a higher maximum canopy conductance, ie plant water demand) would need larger gradients in leaf water potential to sustain the higher fluxes. Thus, based on soil-plant hydraulic prediction, we expect such plants to show a more pronounced TR sensitivity to high VPD (Fig. 1b, Oren et al. 1999; Ranawana et al. 2021; Koehler et al. 2024). However, plant water use responses under elevated VPD are collectively shaped by interactions and trade-offs among these traits (López et al. 2021; Dayer et al. 2022). It is unclear if and how these trait shaping water supply (plant hydraulic conductance) and water demand (canopy conductance) are coordinated with each other. As a result, the combined effect of these traits on plant water use responses to rising VPD remains unclear. Thus, our third objective was to investigate how differences in plant hydraulic traits translate into changes in water use patterns among the set of wheat cultivars released over a century of breeding.

## Material and methods

### Field trial and grain yield measurements

The field experiments to estimate spring wheat (*Triticum aestivum* L.) yield genetic gains were conducted during the 2024 and 2025 growing seasons at the University of Minnesota Agricultural Experiment Station in St. Paul, MN. The 2024 trial was located at 44°59′54.15″ N, 93°10′9.11″ W, and the 2025 trial at 44°59′27.8″ N, 93°10′46.5″ W. The soil at both sites is classified as Waukegan silt loam (Typic Hapludoll, mesic temperature regime).

The cultivars were planted on 14 May 2024 and 17 April 2025, and harvested on 19 August 2024 and 6 August 2025, respectively. The experiment included 29 spring wheat cultivars released from the University of Minnesota wheat breeding program between 1915 and 2022 (Table S1). The criterion for the selection of the specific genotypes was to maximize the coverage of the period of wheat yield increases in that region. A randomized complete block design with three replicates was used each year. Plots consisted of seven 2.4 m rows spaced 15.2 cm apart. Fertilization, weed control, and overall crop management followed standard regional recommendations and were consistent across years. Seasonal weather conditions were recorded on site. Mean daily air temperature during the growing season averaged 19.8 ± 0.3 °C in 2024 and 18.0 ± 0.6 °C in 2025. Total precipitation was 527 mm in 2024 and 496 mm in 2025. These conditions were broadly consistent with the site's long-term averages (Fig. S2). At physiological maturity, all plants within each plot were harvested at ground level, and grain yield was determined after drying samples to a constant weight.

### Characterizing TR responses to VPD

#### Plant material and growth conditions

We selected a subset of 14 genotypes from the cultivars described above that span the full temporal range of release, providing an approximately even representation across years of release (Table S1) for an in-depth characterization of TR response curves to increasing vapor pressure deficit (VPD). Three replicate plants per genotype were seeded at a depth of 2.5 cm in custom-made 4.8 L PVC pots (diameter: 15 cm, height: 30 cm) that had small holes drilled at the bottom to allow for drainage. The pots were filled with 6 kg of dried and sieved (2 mm) haplic luvisol (slightly clayey loam) excavated from an agricultural field in Freising (Germany, 48°24′23.7″N, 11°41′24.4″E) with physical, chemical, and hydraulic properties detailed in Koehler et al. (2023a). Before sowing, slow-release fertilizer was applied at a rate of 5 g per pot (Hauert Tardit Universal 15-7-14-1,5 N-P-K-Mg) and the soil was saturated overnight and initially fertilized with 4.8 g of liquid fertilizer per pot (Gesal Universaldünger 7-5-6 N-P-K). In each pot, four seeds were initially sown and thinned after germination to one seedling per pot, twelve days after sowing (DAS). During growth, the plants were fertilized three more times (15, 21, and 29 DAS) with 0.3, 0.4 and 1.0 g of the liquid fertilizer, respectively. Plants were watered every 1 to 2 days based on the average amount of water that was lost due to evapotranspiration between consecutive days.

All of the 42 plants were grown for 39 days under controlled environment conditions in a growth chamber (Kälte3000 AG, Landquart, Switzerland) under optimal water supply, until 50% or more of the plants reached the stem elongation stage (Zadoks stages: 29 to 31). Environmental conditions during the growth period were as follows: photoperiod: 14 h, photosynthetically active radiation: 362 µmol m^−2^ s^−1^ (light source: LED), measured at canopy height, temperature (night/day): 18/28 °C and VPD 0.8/2.1 kPa. Temperature and VPD data were recorded every 10 min by means of three data loggers (EasyLog, EL-USB-2-LCD, Lascar Electronics, Whiteparish, UK) placed at canopy height at three different positions in the growth chamber. The position of the pots was randomly shuffled every seven days to avoid microclimatic impacts on plant growth.

#### Experimental procedure for establishing TR response curves to increasing VPD

On the evening preceding the increasing VPD exposure, all the pots were watered progressively to dripping and allowed to drain overnight to ensure maximum pot water holding capacity at the onset of the measurements. During that time, the soil surface was covered with a 2 to 3 cm coarse-sized gravel layer and aluminum foil to minimize soil evaporation. The following morning, soil water content (θ, vol.-%) was measured by vertically inserting a Time Domain Reflectometer (5 cm rod length, SM150T, version 4.0, UP GmbH, Ibbenbüren, Germany) into the soil close to the center of the pot to get a measure of soil moisture in proximity to plant roots.

On that same morning, all plants were subjected to a sequence of 6 increasing air VPD levels that were achieved by progressively increasing temperature and lowering RH over two consecutive days (Fig. S3, Table 1), following Schoppach and Sadok (2012) with modifications. We targeted slightly higher VPDs per VPD level on the second day to achieve twice the amount of TR-VPD measurements. Briefly, on each day, each VPD level was maintained for 110 min, and once the transition to the next VPD level was achieved, an acclimation phase of 30 min was allowed for the TR to achieve a steady state (Tamang et al. 2019). At each VPD step, pots were weighed a first time at the end of the 30 min acclimation period and then 60 min afterwards. Weighing took 20 min each time and was performed using a calibrated balance with 0.1 g resolution (Model CPA34001S, Sartorius, Göttingen, Germany). At the end of the increasing VPD exposure, soil water content was measured once more to confirm the absence of soil water limitations (see Results). Pots were re-watered to ensure that initial water status conditions were the same, across the two consecutive days. The same sequence targeting 6 different VPD levels was repeated on the second day, yielding a total of 12 VPD steps (Fig. S3). At the end of the VPD sequence of the second day, whole-plant leaf area (LA, cm^2^) was measured destructively by means of a leaf area meter (LI-3100C; Li-Cor, Lincoln, NE, USA). This was needed to account for genotypic variation due to differences in canopy size in estimating whole plant TR (mg H_2_O m^−2^ s^−1^). The total cumulative TR (TR_tot_, mg m^−2^ day^−1^) was calculated as the sum of TR at the individual VPD levels. Total water loss at the plant level (TWL, ie not normalized by LA, mg day^−1^) was calculated as the sum of pot water loss per VPD level. Roots, stems and leaf blades were harvested and dried at 105 °C for 5 to 8 d to a constant weight. Specific leaf area (SLA, cm^2^ g^−1^) was calculated as the ratio between LA and leaf blade dry mass. Transpiration efficiency (TE, mg_biomass_ mg_water_^−1^) was calculated by dividing the total dry biomass by TWL.

**Table 1:** Temperature (T), relative humidity (RH), and vapor pressure deficit (VPD) conditions that the plants were subjected during the measurements of whole-plant transpiration rates (TR).

	T (°C)		RH (%)		VPD (kPa)	
VPD level	Day 1	Day 2	Day 1	Day 2	Day 1	Day 2
1	19.81	20.72	78.21	78.93	0.49 ± 0.02	0.5 ± 0.01
2	22.86	23.85	58.55	60.54	1.12 ± 0.02	1.14 ± 0.01
3	26.21	26.97	61.5	55.92	1.28 ± 0.02	1.54 ± 0.01
4	29.21	30.51	53.43	51.71	1.87 ± 0.06	2.1 ± 0.01
5	32.26	32.96	43.4	44.37	2.74 ± 0.03	2.81 ± 0.01
6	35.12	35.93	40.95	42.25	3.41 ± 0.04	3.51 ± 0.01

#### Characterizing plant hydraulic conductance

The soil-plant hydraulic conductance (K_sp_, mg m^−2^ s^−1^ kPa^−1^) was estimated on the second day, based on the ratio between TR and the differences (Δ) in leaf water potential (Ψ_leaf_, kPa) across two levels of water fluxes, based on Darcy's law (Eq. 1).


(1) $$K_{\mathrm{sp}}=\frac{TR}{\Delta \psi_{\mathrm{leaf}}}$$

The low-flux measurements were conducted pre-dawn (5:30 AM, before lights were turned on), that is, when TR is minimal and Ψ_leaf_ is almost in equilibrium with the soil water potential (Ψ_soil_) and the high flux measurements were conducted at a VPD of 2.10 ± 0.01 kPa when TR was still increasing linearly with rising VPD (based on the measurements of the previous day).

Considering that the measurements of TR and Ψ_leaf_ were conducted under non-limiting soil water supply, the soil-plant hydraulic conductance is mainly limited by the plant's ability to channel water (Draye et al. 2010). Therefore, K_sp_ is a measure of the plant hydraulic conductance (K_plant_, mg m^−2^ s^−1^ kPa^−1^). Leaf water potentials were measured using a Scholander pressure chamber (Model 3115 Portable Plant Water Status Console, Soilmoisture Equipment Corp., Santa Barbara, CA, USA) on the youngest fully developed leaf of the main stem. Leaves of all plants were collected at the same time and stored in black plastic bags that were equipped with a moist paper towel to avoid a drop in Ψ_leaf_ (Knipfer et al. 2020; Rodriguez-Dominguez et al. 2022). The remainder of the cut leaf was conserved in ethanol for stomatal density measurements. TR refers to the weight loss between start and end time of the respective VPD levels, normalized by leaf area (VPD level 0 and 3, Fig. S3).

#### Nighttime TR measurements

Nighttime TR (TR_night_) was determined gravimetrically (see above) under predawn conditions (5:30 AM and 6:30 AM), before the lights were turned on. The ratio of TR_night_ to daytime TR (TR_day,max._) was calculated by dividing TR_night_ by TR_day,max._, with TR_day,max._ being the daytime TR at the highest VPD level.

#### Stomatal density measurements

Stomatal density was obtained from epidermal peels of the adaxial leaf surface of the youngest fully developed leaf of the main stem per plant using the nail polish method (Pathoumthong et al. 2023). Note that, while wheat has amphistomatous leaves with stomata on both leaf sides, we focused on the adaxial leaf side as it has recently been demonstrated to exhibit a higher stomatal density and a greater contribution to leaf-level gas exchange than the abaxial leaf surface (Wall et al. 2022). However, we acknowledge that measuring stomatal density on both surfaces could provide a more comprehensive understanding of the role of stomatal morphology on leaf-level gas exchange. In terms of the sampling procedure, four 1 cm^2^ segments per leaf were cut in the center of the leaf left and right of the vein. The leaf segments were covered with transparent nail polish and the nail polish was left to dry, after which they were covered with transparent tape. The tape was then transferred onto a microscope slide. Three images per leaf segment were taken with a microscope camera (AxioCam ERc 5 s) at 10× magnification over an area of 0.56 mm × 0.75 mm. For each image, number of stomata was counted using Stomata Counter spot tool (Fetter et al. 2019).

### Data analysis

#### Quantifying changes in grain yield with year of release

To detect potentially nonlinear trends in grain yield (GY) with increasing year of release (YOR), grain yield of the 14 genotypes common to both the controlled-environment and field experiments was regressed against YOR using linear and segmented linear regression models implemented in the R package segmented (version 1.6-4, Muggeo 2003). The linear model was defined as:


(2) $$\mathrm{GY}=GY_{\mathrm{intercept}}+(\mathrm{Slope}\;*\;\mathrm{YOR}).$$

The segmented linear regression was defined as:


(3) $$\mathrm{GY}=(\mathrm{Slop}e_{1}\;*\;\mathrm{YO}R_{\mathrm{BP}}+GY_{\mathrm{intercept}1})+\mathrm{Slop}e_{2}\;*\;(\mathrm{YOR}-\mathrm{YO}R_{\mathrm{BP}}),$$

where Slope_1_, YOR_BP_, and GY_intercept1_ represent the slope of the first linear segment, the estimated breakpoint year of release (YOR_BP_) at which the rate of grain yield increase changed, and the intercept of the first segment, respectively. Slope_2_ represents the slope of the second segment, after YOR_BP_. This method is based on an iterative procedure for detecting a breakpoint, testing for significant differences between the slopes of the two linear segments (Motulsky 2007). If this is the case, the segmented regression modality was retained; otherwise, the linear regression was fitted. The best-fitting model was selected based on an extra sum-of-squares F test (*P* < 0.05).

#### Parameterizing the TR response to increasing VPD

A linear or segmented linear regression was fitted per genotype across replicates using the same procedure as described above in 2.3.1 (Sadok and Sinclair 2009; Monnens et al. 2024). The linear model was defined as:


(4) $$\mathrm{TR}=TR_{\mathrm{intercept}}+(\mathrm{Slope}\;*\;\mathrm{VPD}).$$

The segmented linear regression was defined as:


(5) $$\mathrm{TR}=(\mathrm{Slop}e_{1}\;*\;\mathrm{VP}D_{\mathrm{BP}}+TR_{\mathrm{intercept}1})+(\mathrm{Slop}e_{2}\;*\;(\mathrm{VPD}-\mathrm{VP}D_{\mathrm{BP}})),$$

where Slope_1_, VPD_BP_, and TR_intercept1_ represent the slope of the first linear segment, the breakpoint in air VPD (VPD_BP_) at which the slope in TR-VPD changes, and the intercept of the first segment, respectively. Slope_2_ represents the slope of the second segment, above the VPD_BP_. For each genotype, coefficient estimates and standard errors for the slope of the first linear segment or the linear regression slope (Slope_1_), the VPD_BP_, and the slope difference before and after the VPD_BP_ (in case of a segmented linear regression, slope_diff_) were obtained.

We interpret (i) Slope_1_ (ie TR below the VPD_BP_) as an indicator of stomatal sensitivity to mild increases in VPD or even as reflecting the maximum canopy conductance (Jafarikouhini et al. 2022; Koehler et al. 2024), (ii) VPD_BP_ to indicate the VPD upon which stomatal closure becomes effective in compensating for water loss driven by increasing VPD, thereby initiating restrictions in TR, and (iii) the difference between Slope_1_ and Slope_2_ (Slope_diff._) to be an indicator of the “strength” of the stomatal response (closure) during rising VPD.

#### Characterizing trait variability across genotypes

Genotypic variability TR response curves to increasing VPD was tested using permutational multivariate analysis of variance, treating each VPD level as individual variable (PERMANOVA, Anderson 2001). PERMANOVA was computed on the Manhattan distance for each pair of plants calculated for the TR at each VPD-level. We included the genotype's year of release (YOR) as fixed factor, the day of the experiment as random factor, and their interaction (day:YOR). Each VPD level was treated as a variable. The benefit of analyzing TR∼VPD “multivariately” is that the non-linearity and the non-independence of the measurements can be accounted for in contrast to in an ANOVA or ANCOVA analysis. The PERMANOVA was conducted in PRIMER version 7.0.23.

Genotypic differences between plant morphological [leaf area (LA), leaf blade dry mass, specific leaf area (SLA), root biomass, root:shoot ratio, stomatal density], and hydraulic traits (K_plant_) were tested according to the following steps. First, the Bartlett Test of Homogeneity of Variances was conducted (bartlett.test function in R version 4.1.2). If the variances in each of the groups were the same (*P*-value > 0.05), an ANOVA was conducted (aov function in R version 4.1.2). Subsequently, the residuals of the ANOVA-outcome were tested for normal distribution using the Shapiro-Wilk Normality Test (shapiro.test function in R version 4.1.2). If the residuals were normally distributed (*P*-value > 0.05), the Tukey Honest Significant Differences—test was applied post-hoc to identify the groups that differed significantly (TukeyHSD function in R version 4.1.2). If the variances in each of the groups were not the same (Bartlett Test of Homogeneity of Variances *P*-value < 0.05) or the residuals of the ANOVA-outcome were not normally distributed (Shapiro–Wilk Normality Test *P*-value < 0.05), a Kruskal–Wallis Rank Sum Test was conducted (kruskal.test function in R version 4.1.2), followed by Dunn's Test (post hoc) in order to identify the groups that differed significantly (dunn.test function in R version 1.3.5).

#### Characterizing trait association with of release

**Figure 2:**
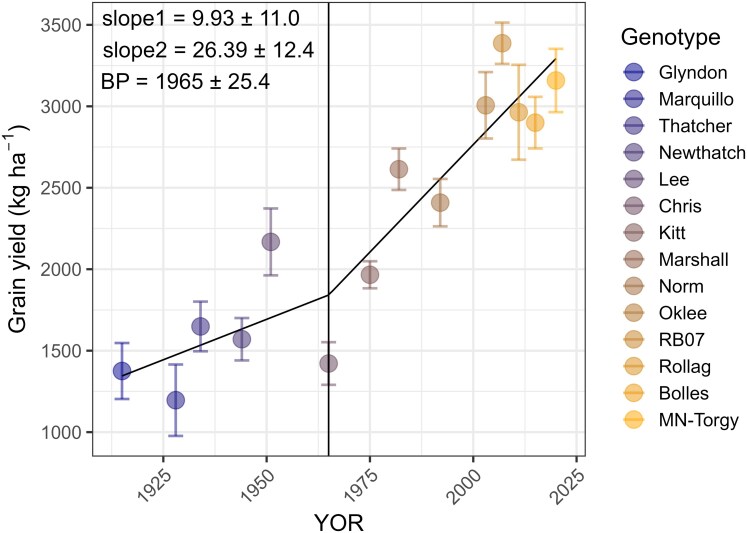
Grain yield per year of release (YOR) of the here tested genotypes.

Because our data analysis of yield genetic gains identified a nonlinear increase in grain yield with increasing year of release, with an uptick at 1965 (±25.4 years, Fig. 2), we used that year as a cut off for further analyses.

Linear and segmented linear regressions, following the same procedure described above, were applied to relationships between YOR and (i) parameters derived from the TR–VPD response curves and (ii) morphological and hydraulic traits. When segmented regressions identified a breakpoint, it was retained if it occurred within the 1965 ± 25.4-year window, corresponding to the breakpoint detected for grain yield. Otherwise, a linear regression was applied. Note that the slope indicated in Fig. 5 refers to standardized slopes after the breakpoint resulting from standardizing both trait values and yield (mean-centered and scaled to unit variance) prior to regression to allow for a direct comparison of effect sizes across traits differing in scale and units.

#### Correlations between grain yield and hydraulic and developmental traits

To establish correlations between grain yield and traits measured, we conducted linear regressions between those variables for genotypes released after the nonlinear change in grain yield, 1965 ± 25, ie when genetic gains in grain yield were at their highest rate of increase. Both trait values and yield were standardized (mean-centered and scaled to unit variance) prior to regression to obtain standardized slopes. This allowed effect sizes to be directly comparable across traits differing in scale and units.

#### Correlations between plant water use and hydraulic traits

To assess relationships between indicators of plant water use under high evaporative demand (ie VPD_BP_, Slope2) and hydraulic traits that are expected to regulate those responses (ie K_plant_, Slope1, Fig. 1), linear regression analyses were performed for all variable combinations. Because the dataset contained influential observations (identified based on Cook's distance), analyses were conducted both with and without these points. In the main text, as well as in Fig. 7 and Fig. S5, we report *P*-values and R^2^ values from models in which influential observations were accounted for (ie excluded). Results obtained without excluding influential points are provided in Fig. S6.

### Conceptual model

In order to relate TR-VPD response to plant hydraulic traits controlling for plant water supply (ie the plant hydraulic conductance, K_plant_) and plant water demand (ie the maximum canopy conductance, g_c max._) in Fig. 1, we used the soil-plant hydraulic model of Carminati and Javaux (2020). The model was implemented as in Wankmüller and Carminati (2022) and applied as in Koehler et al. (2023b) by modifying the parameters in Table 2. Briefly, the model by Carminati and Javaux (2020) assumes that stomatal regulation is related to limitations in the soil-plant hydraulic conductance, which determines the water supply function. Exceeding this water supply function manifests in a nonlinear relationship between TR and leaf water potential at a given soil moisture. This nonlinearity is inconvenient for plants as a slight increase in TR requires a disproportional decrease in leaf water potential. Therefore, stomata are expected to respond to this nonlinear change in TR in relation to a change in leaf water potential by partially closing, thereby lowering the TR and softening water potential gradients. The model calculates the leaf water potentials based on TR, soil matric potential, and the hydraulic conductances of the components of the soil-plant-atmosphere-continuum (a detailed description of the model can be found in Carminati and Javaux 2020). The resulting leaf water potentials serve as input for the stomatal regulation model as added by Wankmüller and Carminati (2022). The relationship between leaf water potential and gas exchange informs the stomatal regulation model, which is based on optimizing the carbon assimilation rate to leaf water potential ratio. Note that, in essence, the stomata model follows the same assumptions as the one by Cowan and Farquhar (1977), with the difference that the water cost is described in terms of water potential and not in terms of water flux. Notably, the two versions (water potential versus water flux) are equivalent as long as the relationship between flux and potential is linear, which is the criterion for the onset of water limitation defined in Carminati and Javaux (2020).

**Table 2:** Modified parameter setting to run the soil-plant hydraulic model after Carminati and Javaux (2020) as implemented in Wankmüller and Carminati (2022).

Parameter	Abbreviation	Value	Unit
Plant hydraulic conductance	K_plant_	[1.49 × 10^−7^, 1.25 × 10^−7^, 1.01 × 10^−7^, 7.75 × 10^−8^, 5.37 × 10^−8^, 2.98 × 10^−8^]	cm^3^ s^−1^ hPa^−1^
Maximum stomatal conductance	g_c max._	[191.74, 213.21, 237.08, 263.62, 293.14, 325.96]	mmol m^−2^ s^−1^
Saturated soil hydraulic conductivity	k_sat_	4.39 × 10^−4^	cm s^−1^
Effective root length	L	5,000	cm
Vapor pressure deficit	VPD	[0.1, 4.5]	kPa

Parameters not listed here were used as per the default parameter setting in Wankmüller and Carminati (2022).

## Results

### Increase in yield genetic potential as a function of the year of release

A first requirement for this study was that the studied genotypes capture genetic gains in yields as proxied by their years of release (YOR). As shown in Fig. 2, grain yield of the tested genotypes increased by a factor of 2.5 between 1915 and 2022 and the genotypic variability for yield was highly significant (F(13, 70) = 18.30, *P* < 0.001). Furthermore, this increase appears to be nonlinear, showing an increase in the rate of genetic yield gains starting from 1965 ± 25.4. Prior to this year, the rate of yield increase was 9.93 kg ha^−1^ yr^−1^ while it rose to 26.39 kg ha^−1^ yr^−1^ afterwards. This result confirms the relevance of the cultivars’ selection in examining the association between yield potential and the physiological parameters considered in this study.

### Requirements for constructing TR response curves to rising VPD

To examine the effect of increasing VPD on TR, two conditions had to be met. Firstly, VPD conditions should be stable at each VPD step so that TR can be assumed to be at steady state. As shown in Fig. S3, VPD levels remained constant at each step across days. Consistently with this, genotype-specific TR responses to increasing VPD were not significantly different across days (PERMANOVA, pseudo-F = 0.41, *P* = 0.98, Table S2), indicating that the VPD treatment yielded consistent and repeatable TR responses.

**Figure 3:**
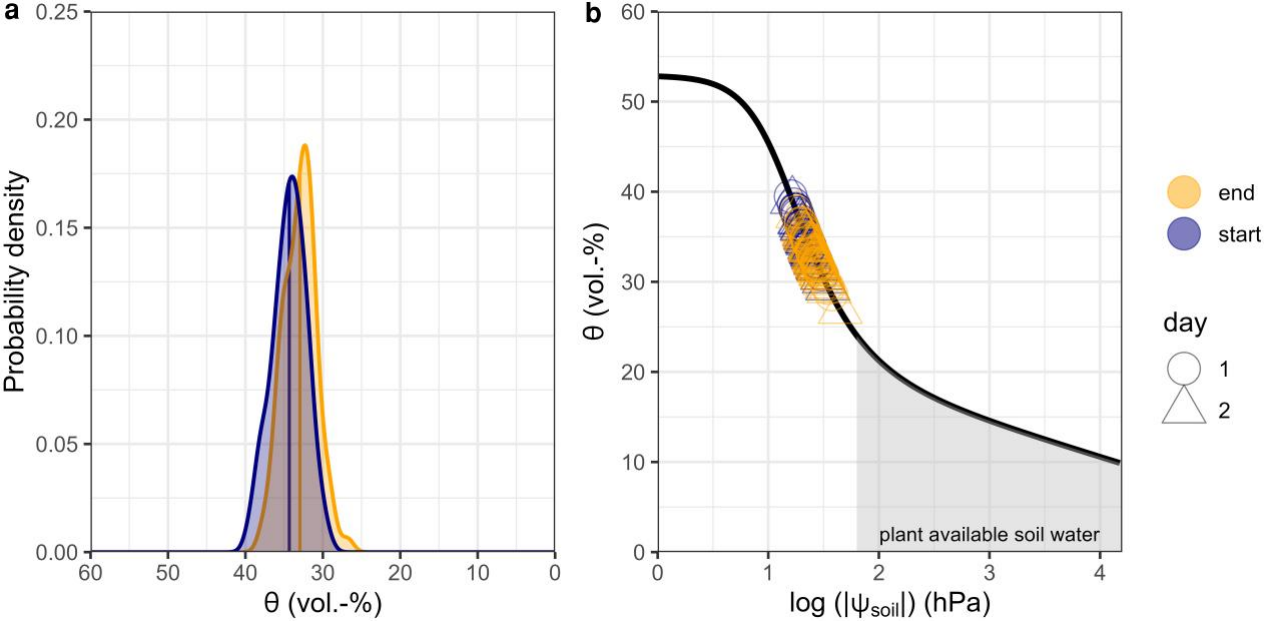
a) Probability density function of soil water content (θ) as measured for each pot in the beginning of the day and at the end of the day after the exposure to increasing VPD. Vertical lines mark the average soil water content at both time points. b) Soil water retention curve (soil water content, θ vs. soil water potential, Ψ_soil_) of the used soil material with water contents as measured in the beginning of the day and at the end of the day marked along the curve for both days (circles—day 1, triangles—day 2). The range of theoretically plant available soil water in the typical range between 60 and 15,000 hPa is marked by the gray-shaded area.

Secondly, VPD effects on TR should be independent from soil moisture deficit. As shown in Fig. 3a, while bulk soil water content (θ) decreased significantly from an average of 34.3 ± 0.24 vol.-% (onset of the first VPD step) to 33.0 ± 0.23 vol.-% (end of the last VPD step) due to TR (t(83) = 5.70, *P* = <0.01), all plants were still having access to freely available soil moisture (ie soil water potential > −0.0016 MPa, Fig. 3b).

### Genetic variability in TR response to rising VPD among cultivars

**Figure 4:**
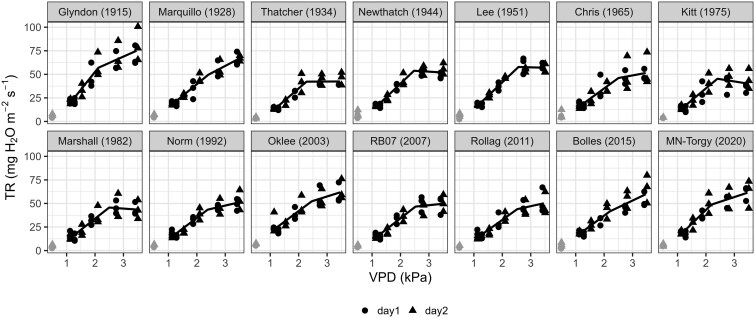
Transpiration rate (TR) response curves to increasing vapor pressure deficit (VPD) as measured on two consecutive days (dots = day 1, triangles = day 2) and fitted (lines) by a segmented linear regression across replicates (*n* = 3) per genotype (*n* = 14). Gray symbols indicate nighttime TR measurements. The number reported next to the cultivar name represent the official year of release.

The effect of rising VPD on whole-plant TR was found to be significantly variable across genotypes (PERMANOVA, pseudo-F = 10.54, *P* < 0.01, Table S2). Furthermore, all tested varieties exhibited a segmented TR response to rising VPD within the tested range of VPD (1.14 to 3.51 kPa, Fig. 4). Across varieties, Slope1 varied almost 2-fold, ranging from 21.67 to 37.88 mg H_2_O m^−2^ s^−1^ kPa^−1^. The VPD_BP_ ranged from 2.11 to 2.57 kPa. The percentage difference between TR-VPD slopes below and above the VPD_BP_ varied more than 3-fold between 36% and 119% (Table 3).

**Table 3:** Segmented regression parameters of transpiration rate (TR) response curves to rising vapor pressure deficit (VPD) for each cultivar.

		Slope1 (mg m^−2^ s^−1^ kPa^−1^)		Slope2 (mg m^−2^ s^−1^ kPa^−1^)		Slope_diff._ (%)		VPD_BP_ (kPa)	
Genotype	YOR	Est.	SE	Est.	SE	Est.	SE	Est.	SE
Glyndon	1915	37.88	6.44	13.45	10.43	64.49	27.53	2.11	0.40
Marquillo	1928	24.76	3.88	15.75	6.28	36.41	25.36	2.40	0.60
Thatcher	1934	26.02	5.52	0.16	8.93	99.38	34.33	2.28	0.31
Newthatch	1944	28.84	5.32	−1.82	8.62	106.29	29.87	2.47	0.24
Lee	1951	28.63	3.98	−0.87	6.44	103.04	22.48	2.57	0.19
Chris	1965	21.96	4.03	5.56	6.52	74.69	29.68	2.50	0.34
Kitt	1975	25.20	5.58	−4.81	9.04	119.09	35.86	2.40	0.26
Marshall	1982	23.70	5.60	−2.19	9.07	109.25	38.25	2.50	0.30
Norm	1992	22.89	7.57	7.03	12.25	69.27	53.53	2.38	0.67
Oklee	2003	22.55	9.50	9.53	15.38	57.75	68.22	2.46	1.01
RB07	2007	23.53	6.82	3.11	11.05	86.76	46.96	2.53	0.46
Rollag	2011	21.67	5.95	6.64	9.64	69.33	44.47	2.52	0.55
Bolles	2015	22.43	3.76	14.41	6.09	35.73	27.17	2.20	0.69
MN-Torgy	2020	29.45	5.83	9.96	9.45	66.19	32.07	2.19	0.44

Overview of coefficient estimates and their standard errors (SE) from the linear and linear segmented regression per genotype: the slope of the first linear segment in case of a segmented regression or the regression slope in case of a simple linear regression (Slope1), the VPD upon which the increase in TR with rising VPD was restricted (VPD_BP_), and the difference in the two slopes of the segmented regression (Slope_diff._).

While cumulative TR varied significantly between genotypes by a factor of 1.6 (TR_tot_, F(13,28) = 2.60, *P* = 0.02), total plant water loss (TWL) showed no significant genotypic variability (χ^2^ (13, N = 42) = 12.00, *P* = 0.53), as well as transpiration efficiency (TE), (F(13,28) = 0.67, *P* = 0.77). Nighttime TR (TR_night_) varied significantly (χ^2^ (13, N = 42) = 24.51, *P* = 0.03) across genotypes, between 3.95 and 6.57 mg m^−2^ s^−1^. None of the tested genotype exhibited a zero TR_night_, and in fact, the ratio of TR_night_ to maximum daytime TR measured at the highest VPD, averaged 10%, with no significant genotypic differences (F(13,28) = 0.85, *P* = 0.61). Leaf area (LA, F(13,28) = 1.35, *P* = 0.25, average: 1,063.73 ± 27.24 cm^2^), specific leaf area (SLA, F(13,28) = 1.69, *P* = 0.12, average: 244.73 ± 4.08 cm^2^ g^−1^) and leaf blade dry mass (F(13,28) = 1.06, *P* = 0.43, average: 4.35 ± 0.09 g) did not vary significantly between genotypes as well as root dry biomass (F(13,28) = 0.79, *P* = 0.66, average: 5.91 ± 0.35 g) and root:shoot dry biomass ratio (F(13,28) = 0.92, *P* = 0.55, average: 0.80 ± 0.04 g g^−1^). In contrast, plant hydraulic conductance (K_plant_, F(13,28) = 2.39, *P* = 0.03, average: 0.40 ± 0.05 mg m^−2^ s^−1^ kPa^−1^) and stomatal density (F(13,28) = 4.03, *P* = <0.01, average: 82.51 ± 2.62 per mm^2^) varied significantly between genotypes (Table S3).

### Trait association with year of release

**Figure 5:**
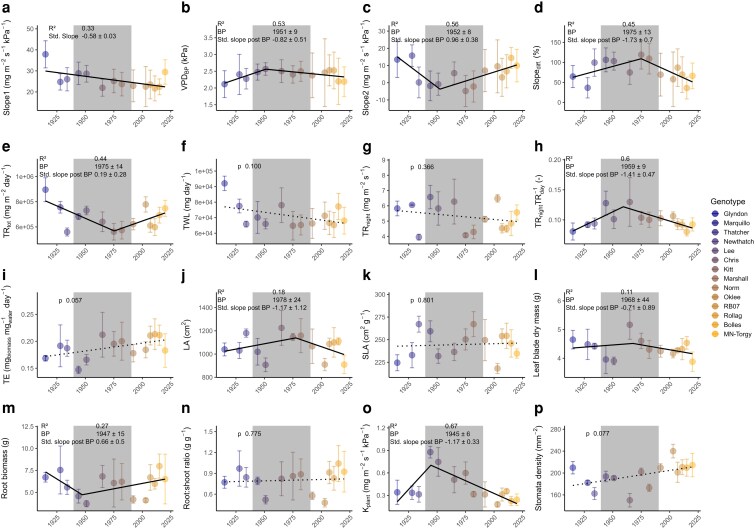
Association between measured traits and the year of release (YOR) of the tested genotypes: a) the initial slope of the TR-VPD response (Slope1), b) the VPD upon which the slope in TR changed with rising VPD (VPD_BP_), c) the slope after the VPD_BP_ (Slope2), d) the percentage difference in slope before and after the VPD_BP_ (Slope_diff._), e) the total daily cumulated transpiration rate (TR_tot_), f) the total water loss over the course of the day at the whole plant level (ie without normalizing by leaf area, TWL), g) nighttime transpiration rate (TR_night_), h) ratio between nighttime and daytime transpiration rate at the highest imposed VPD level (TR_night_ TR_day_^−1^), i) transpiration efficiency (TE), j) leaf area (LA), k) specific leaf area (SLA), l) leaf blade dry mass, m) root biomass, n) root:shoot ratio, o) plant hydraulic conductance (K_plant_), and p) stomatal density. Error bars represent standard errors of the model-derived parameters (a–d) and standard errors of the mean for each trait-genotype combination (e–p). A dotted regression line indicates the absence of a statistical association between measured traits and genotypes with increasing YOR (*P* > 0.05). Std. slope refers to standardized slope (after the breakpoint YOR, in the case of a segmented regression) resulting from standardizing both trait values and YOR (mean-centered and scaled to unit variance).

Among the investigated parameters, only the first slope of the TR-VPD relation (Slope1) exhibited a significant linear change with increasing year of release (YOR), decreasing by 0.072 mg m^−2^ s^−2^ kPa^−1^ year^−1^ (Fig. 5a).

Based on the nonlinearity in yield trends that we observed, we further examined potential nonlinear changes in these traits and TR responses within the same time window during which the cultivars tested here exhibited the breakpoint leading to the uptick in the rate of genetic yield gains (1965 ± 25.4 years, Fig. 2).

For the water use response traits, the analysis revealed that VPD_BP_ decreased slightly but significantly by 0.0034 kPa year^−1^ after 1951 (Fig. 5b), while Slope2 increased by 0.21 mg m^−2^ s^−1^ kPa^−1^ year^−1^ after 1952 (Fig. 5c), reflecting a significant reduction in the difference between Slope1 and Slope2 by 1.3 % year^−1^ (Fig. 5d), that is, a progressive linearization of the TR–VPD relationship. This was the strongest observed change across the dataset (standardized absolute slope after the critical YOR = 1.73). Consequently, leaf level cumulative daily water loss increased after 1975 by 3201 mg m^−2^ d^−1^ yr^−1^ (Fig. 5e). This is also reflected in the decrease in TR_night_ TR_day_^−1^ after 1959 by 0.0006 yr^−1^ (Fig. 5h), considering that TR_night_ did not change significantly (Fig. 5g) and a decrease in TR_night_ TR_day_^−1^ is likely related to an increase in TR_day_. This was the second strongest observed change across the dataset (standardized absolute slope after the critical YOR = 1.41). A non-significant increasing trend in TE with YOR (*P* = 0.057, Fig. 5i) suggests a tendency toward improved water-use effectiveness. This pattern likely reflects the coordinated changes in multiple water-use traits, indicating the emergence of an integrated water-use strategy that collectively contributes to higher TE.

In the case of the hydraulic and developmental traits, LA (Fig. 5j) and leaf blade dry mass (Fig. 5l) decreased significantly after 1978 and 1968 by 3.4 cm^2^ yr^−1^ and 0.0069 g yr^−1^, respectively, compensating for the increase in cumulative TR (Fig. 5e), and leading to nonsignificant changes in total plant water loss (Fig. 5f). While root dry biomass (Fig. 5m) increased by 0.0245 g yr^−1^ after 1947, K_plant_ showed a steep decrease by 0.00679 mg m^−2^ s^−2^ kPa^−1^ yr^−1^ after 1945 (Fig. 5o). This was the third strongest observed change across the dataset (standardized absolute slope after the critical YOR = 1.17). Plant allometric relationships (eg SLA, root:shoot ratio, Fig. 5k and n) did not change across genotypes released over consecutive years, but stomatal density seems to show a nonsignificant increasing trend (*P* = 0.077, Fig. 5p).

### Trait association with grain yield

**Figure 6:**
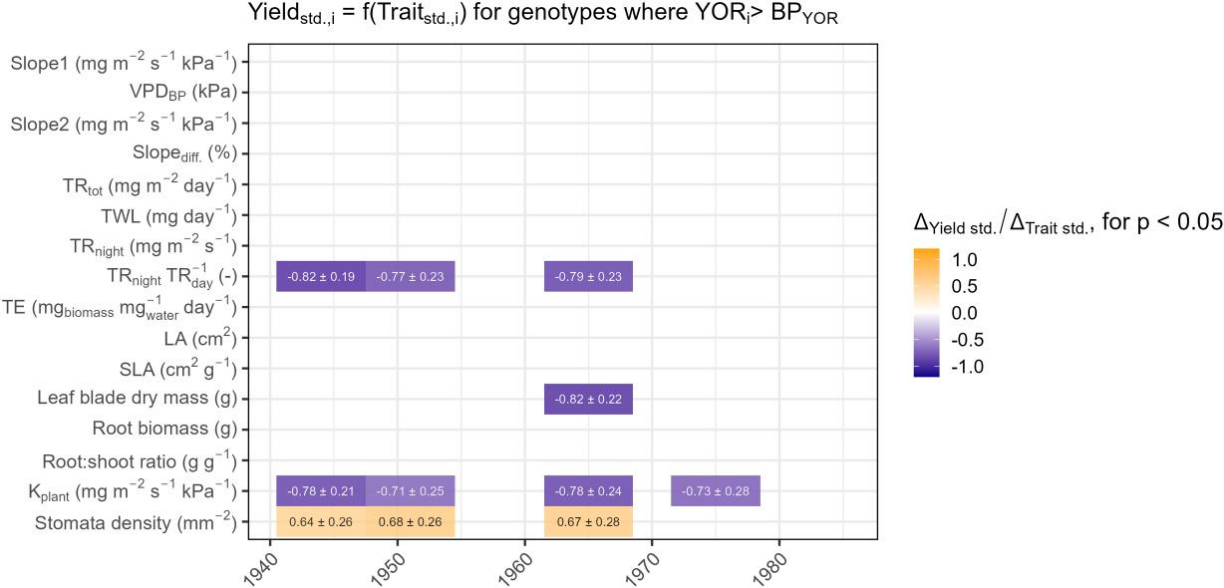
Correlation heatmap between (standardized) grain yield (Fig. 2) and (standardized) trait values (Fig. 5, *y*-axis) after the nonlinear increase in grain yield (1965 ± 25.4 years), considering different starting points in accordance with the standard error for the onset of the yield increase (*x*-axis) for the following traits: the initial slope of the TR-VPD response (Slope1), the VPD upon which the slope in TR changed with rising VPD (VPD_BP_), the slope after the VPD_BP_ (Slope2), the percentage difference in slope before and after the VPD_BP_ (Slope_diff._), the total daily cumulated transpiration rate (TR_tot_), the total water loss over the course of the day at the whole plant level (ie without normalizing by leaf area, TWL), nighttime transpiration rate (TR_night_), ratio between nighttime and daytime transpiration rate at the highest imposed VPD level (TR_night_ TR_day_^−1^), transpiration efficiency (TE), leaf area (LA), specific leaf area (SLA), leaf blade dry mass, root biomass, root:shoot ratio, plant hydraulic conductance (K_plant_), and stomatal density. Blue tiles indicate increasing yield with decreasing trait values, while orange tiles indicate increasing yield with increasing trait values. Tiles are only shown if the association between yield and traits was significant (*P* < 0.05).

Varieties released after 1965 ± 25.4 years, ie “Green Revolution” cultivars, exhibited several trait-yield associations (Fig. 6). These reflected a negative association of TR_night_ TR_day_^−1^, leaf blade dry mass, and K_plant_ with yield increase and the opposite trend for the association between stomatal density and yield (Fig. 6).

### Associations between plant water use and hydraulic traits

**Figure 7:**
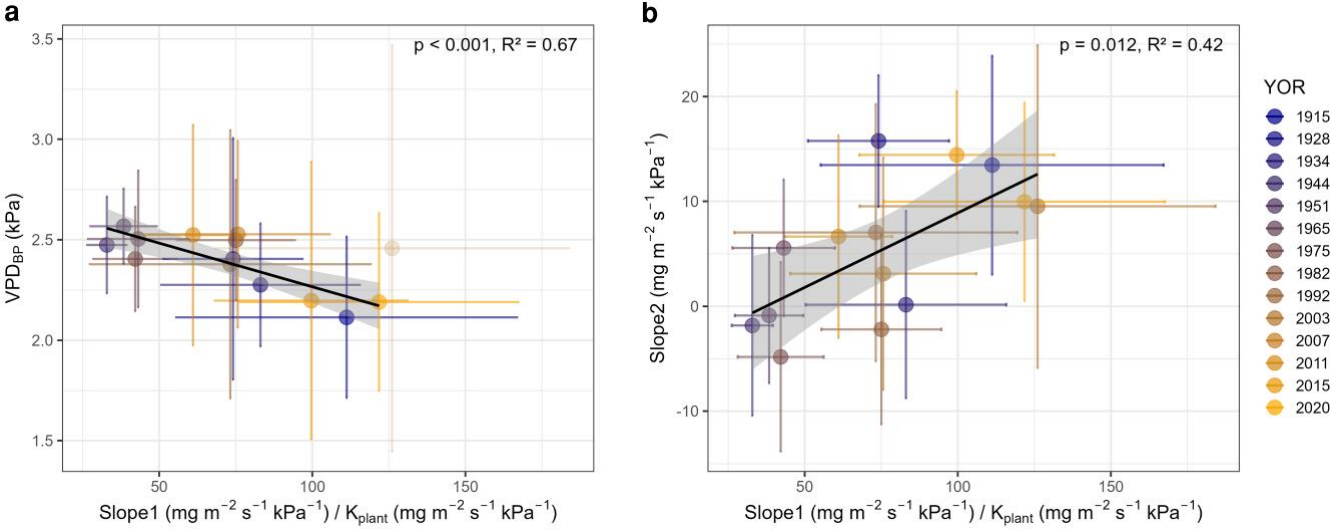
Relationship between (a) the VPD at which the increase in the transpiration rate was restricted with rising VPD (the VPD breakpoint, VPD_BP_) and the ratio between the maximum canopy conductance (Slope1, ie proxy for g_c max._) and the plant hydraulic conductance (K_plant_), (b) the slope after the VPD_BP_ (Slope2) and the ratio between the maximum canopy conductance (Slope1, ie proxy for g_c max._) and the plant hydraulic conductance (K_plant_). Error bars represent standard errors of the model-derived parameters (Slope1, VPD_BP_, Slope2) and standard errors of the mean of K_plant_ for each genotype. Influential observations (identified based on Cook's distance) were accounted for in all analyses. These points are displayed with increased transparency: (a) 2003 (Oklee), (b) none. Not accounting for these influential points leads to the same pattern (*P* = 0.011, *R*^2^ = 0.43, Fig. S6a).

Among the 14 varieties, plants with a comparatively higher water demand, indicated by Slope1 (used here as a proxy of maximum stomatal conductance, g_c max._), relative to their water supply, characterized by whole-plant hydraulic conductance (K_plant_) tended to initiate restrictions in TR at lower VPD as reflected by lower VPD_BP_ (Fig. 7a). The individual effects of K_plant_ and Slope1 on VPD_BP_ were less pronounced (Fig. S5a, b).

In contrast, plants exhibiting a high Slope1 relative to K_plant_ tended to display a lower relative decrease in canopy conductance in response to increasing VPD beyond the VPD_BP_ (ie higher Slope 2, Fig. 7b). Accordingly, both K_plant_ and Slope1 appeared to exert negative effects on Slope2 (Fig. S5d, e).

## Discussion

### Wheat breeders in the Upper Midwest have reinforced conservative plant water use, while reducing VPD sensitivity

A first major finding of this work is that all 14 tested genotypes, released over 105 years, exhibited a segmented TR response to increasing VPD within the tested VPD range (Fig. 4) in the clear absence of soil water limitations (Fig. 3). This suggests that breeders have consistently, albeit likely unintentionally, selected for genotypes in which water conservation through stomatal closure becomes effective in compensating for water loss as driven by the increase in VPD, ie genotypes with a pronounced stomatal sensitivity to rising VPD. This aligns with findings from Schoppach and Sadok (2012) and Schoppach et al. (2017), who reported that Australian-bred wheat cultivars adapted to drought-prone environments displayed a limited TR response to rising VPD. Our results suggest that even in less arid regions such as the US upper Midwest, wheat yields remain constrained by VPD. This is consistent with a simulation study by Sinclair et al. (2010), who suggested that US soybean yield increases could be achieved through limiting TR under high VPD even in temperate regions of the USA, and with a recent study by Zhen and Sadok (2026), who suggested the same for spring wheat in the US spring wheat growing region. In those studies, limiting TR was interpreted as a conservative water use strategy, which is debated to be beneficial under terminal drought conditions (Messina et al. 2015; Sinclair et al. 2017). The consistent expression of a restricted TR across years supports the proposed benefits of this phenotype.

Considering changes in breeding technologies that occurred around the Green Revolution (1960/1970), it is not surprising that we detected nonlinearities in developmental trends for grain yield and hydraulic-morphological traits (Figs. 2 and 5). Yet, to our knowledge, such nonlinear patterns have never been reported in previous studies. Specifically, we observed a decrease in the VPD threshold at which stomatal closure begins to limit water loss (VPD_BP_, Fig. 5b) together with a decrease in water loss rates below this threshold (Slope1, Fig. 5a), and an increase in water loss rates above this threshold (Slope2, Fig. 5c) around the same period (1965 ± 25.4 years). Schoppach et al. (2017) have found similar effects for VPD_BP_ and Slope2 in 13 historic Australian wheat cultivars released from 1890 and 2008. The reduced stomatal sensitivity at high VPD (Slope2, Fig. 5c) led to a linearization of the TR-VPD response and higher cumulative TR (TR_tot_, Fig. 5e, h). However, this effect was offset by a simultaneous reduction in evaporative surface area (LA, Fig. 5j, l), resulting in no significant change in total plant water loss (Fig. 5f), contrary to trends reported for breeding-related developments in European wheat varieties (Behrend et al. 2026). While the principle finding of Behrend et al. (2026), that breeding has altered water use in (winter) wheat, is consistent with our results, the authors report lower seasonal water use in modern compared to historic cultivars based on simulations. It remains to be investigated how the changes in daily water-use patterns observed here translate to water use over an entire growing season, and how drivers of field-scale evaporative demand other than VPD jointly influence plant water use in a canopy (Vadez and Pilloni 2025). However, recent simulation evidence from the US spring wheat region suggests that TR sensitivity to VPD is a favorable trait and may further increase yield under future climate scenarios (Zhen and Sadok 2026).

Given that the rate of grain yield increase per year of release nearly tripled during this period, these results suggest that the shift in the timing (earlier VPD_BP_) and regulation of daily water use (higher Slope2) may have promoted a more effective water use (ie increase in carbon assimilation per unit of water transpired, Vadez et al. 2014; Ding et al. 2025), as also suggested by the increasing trend in transpiration efficiency (Fig. 5i, Behrend et al. 2026). Additionally, the increase in total daytime TR (Figs. 5e, h, and 6) combined with reduced investment in vegetative biomass (Figs. 5j, l, and 6) may have contributed to yield gains by allocating assimilated carbon more efficiently to grain production (ie higher harvest index, Siddique et al. 1990; Reynolds et al. 2009; Lorenz et al. 2010). We interpret the increasing trend in stomatal density (Fig. 5p) and its positive association with yield potential following the nonlinear increase in yield potential (Fig. 6) as indicative of a compensatory strategy for reduced evaporative surface area. This is supported by the significant negative relationship observed between stomatal density and leaf area (Fig. S4j). Increased stomatal density may also have contributed to the observed rise in photosynthetic capacity (Xu and Zhou 2008; Tanaka et al. 2013), as recently reported for wheat by Ding et al. (2025). However, it should be noted that the relationship between photosynthesis and stomatal traits is environment-dependent, and photosynthesis, as being the result of a combination of diffusive and biochemical processes, can be limited by stomatal and non-stomatal factors (Yin et al. 2020). To confirm this, follow-up studies should incorporate direct measurements of leaf-level gas exchange to also capture the biochemical component.

Previous work by Tamang et al. (2019), who also examined wheat genotypes adapted to the Midwestern US, showed that only one out of 29 genotypes exhibited a TR sensitivity to VPD within the tested VPD range (1.4 to 3.0 kPa). This discrepancy is likely related to differences in experimental design. In Tamang et al. (2019), VPD was varied while maintaining a constant temperature (30 °C) to avoid temperature-dependent effects on TR. Such temperature-driven processes include changes in membrane permeability, leading to increased stomatal TR, the increase in cuticle permeability, leading to greater cuticular TR, and the decrease in water viscosity due to temperature increase (for a review see Sadok et al. 2021). The observation that the genotypes tested in Tamang et al. (2019) exhibited a linear TR response to VPD while presenting a segmented response in the present study over a similar range in VPD indicates that the hydraulic limitations reported here are likely partially driven by concurrent changes in temperature. To verify this, we propose follow-up studies that focus on disentangling the effect of VPD from the effect temperature by decreasing RH only. If confirmed, our results suggest that covariations in temperature might have led to a more conservative water use than previously recognized. Since temperature and VPD typically increase concurrently under field conditions, the screening approach implemented in this study likely revealed phenotypes that are more representative of those expressed in the field. Additionally, differences in the daytime VPD during growth conditions (Tamang et al. 2019: between 2.4 and 3.2 kPa, here: 2.1 kPa) relative to the range of tested VPD (Tamang et al. 2019: up to 3.3 kPa, here: up to 3.5 kPa) might have generated VPD acclimation effects (Seversike et al. 2013; Riar et al. 2015; Choudhary et al. 2020), which are likely to impact VPD sensitivity (López et al. 2021).

### Plant hydraulic traits shape plant water use during atmospheric drying

This work provides evidence supporting the importance of plant hydraulics in regulating plant water use and productivity in response to atmospheric drying. One key finding is that the VPD at which the initiation of TR restrictions occurred (VPD_BP_) showed a negative trend with the ratio between the maximum canopy conductance (Slope1) and the plant hydraulic conductance (K_plant_, Fig. 7a). In other words, the TR increase with rising VPD was restricted at lower VPD for plants with a high water demand at low VPD and for plants with a limited water channeling ability. This aligns with predictions from simple soil-plant hydraulic principles (Koehler et al. 2024, Fig. 1c, d). Interestingly, the individual effects of K_plant_ and Slope1 on VPD_BP_ were less pronounced (Fig. S5a, b). This may be explained by the opposing effects of K_plant_ and Slope1 on VPD_BP_ (Fig. 1). While K_plant_ is expected to increase VPD_BP_ (Fig. 1a, c), Slope1 exerts a negative effect (Fig. 1b, d). However, because Slope1 and K_plant_ both showed a tendency of being positively associated (Fig. S5c) and both decreased with YOR (Fig. 5a, o), their contrasting influences likely overlapped, thereby partially masking their individual effects on VPD_BP_. In light of these trade-offs and synergies between traits, simulating water use responses to atmospheric drought could benefit from incorporating hydraulics, eg in crop models (Torres-Ruiz et al. 2024). While crop models like SSM (Sadok et al. 2019) and APSIM (Collins et al. 2021) can already account for the effect of TR rate limitations at high VPD on water and carbon budgets (Zhen and Sadok 2026), inferring TR–VPD response curves from measurable and dynamic plant hydraulic traits could provide more realistic, genotype- and environment-specific estimates of the VPD threshold at which TR becomes restricted. Such an approach would enable evaluating previously untested hydraulic traits and processes in predicting yield outcomes in response to rising VPD and thus potentially guide the development of new phenotyping and breeding strategies.

The “intensity” of stomatal closure, quantified here as Slope2, is less frequently discussed as indicative of a water use strategy, yet it plays a significant role in determining daily water consumption and its difference relative to Slope1 changed most prominently over time here (Fig. 5d), which is consistent with Schoppach et al. (2017). Moreover, Slope2 is as well related to hydraulic traits (Fig. 7b, Fig. S5d, e). Restrictions in the transpiration rate with rising VPD beyond the VPD_BP_ were less effective (ie higher Slope2 and a more linear TR-VPD response) for plants with a high initial water demand (ie maximum canopy conductance, Slope1) relative to their water channeling ability (K_plant_, Fig. 7b). Although earlier restrictions in transpiration rate increased water loss rates beyond the VPD_BP_ (Fig. S5f) and total leaf-level water loss (Fig. 5e), this water-use strategy also indicates a trend toward enhanced transpiration efficiency (Fig. 5o). This idea points to the possibility of designing and breeding for highly fine-tuned hydraulic phenotypes to maximize water use efficiency (Zhen and Sadok 2026).

## Concluding remarks

This study revealed nonlinear changes in yield potential of US Midwestern spring wheat around the Green Revolution (1965 ± 25 years) and relates these changes to shifts in daily water-use patterns in response to atmospheric drying, and to associated developmental and hydraulic traits as cryptically fixed through breeding. Overall, breeding maintained a restricted transpirational water loss under high VPD across all cultivars. However, the tripling of yield potential around the Green Revolution coincided with a successive linearization of the TR-VPD response, which nevertheless remained nonlinear. Combined with reduced evaporative surface area in modern cultivars, this resulted in unchanged whole-plant water loss. Given the simultaneous substantial increase in grain yield, this indicates improved efficiency of water-use, rather than a reduction in total water consumption by the crop. Daily water-use regulation was associated with traits governing water demand and supply. TR was restricted at lower VPD, but less effectively (ie higher water loss rates beyond that VPD) in plants exhibiting higher water demand at low VPD relative to their water channeling ability. These findings highlight opportunities to improve yields through targeted adjustment of hydraulic traits and particularly TR sensitivity to VPD.

## Supplementary Material

kiag500_Supplementary_Data

## Data Availability

Data supporting the findings of this study are available within the paper, within its Supplementary data or on request.
