# Supplementary material for: Green revolution breeding favored water conservation, but weakened water use sensitivity to rising vapor pressure deficit in US spring wheat
Source: Plant Physiol. 2026 Jul 14;201(3):kiag500. doi: 10.1093/plphys/kiag500 (PMC13418363; doi:10.1093/plphys/kiag500)
Supplement: kiag500_Supplementary_Data [file kiag500_supplementary_data.docx]

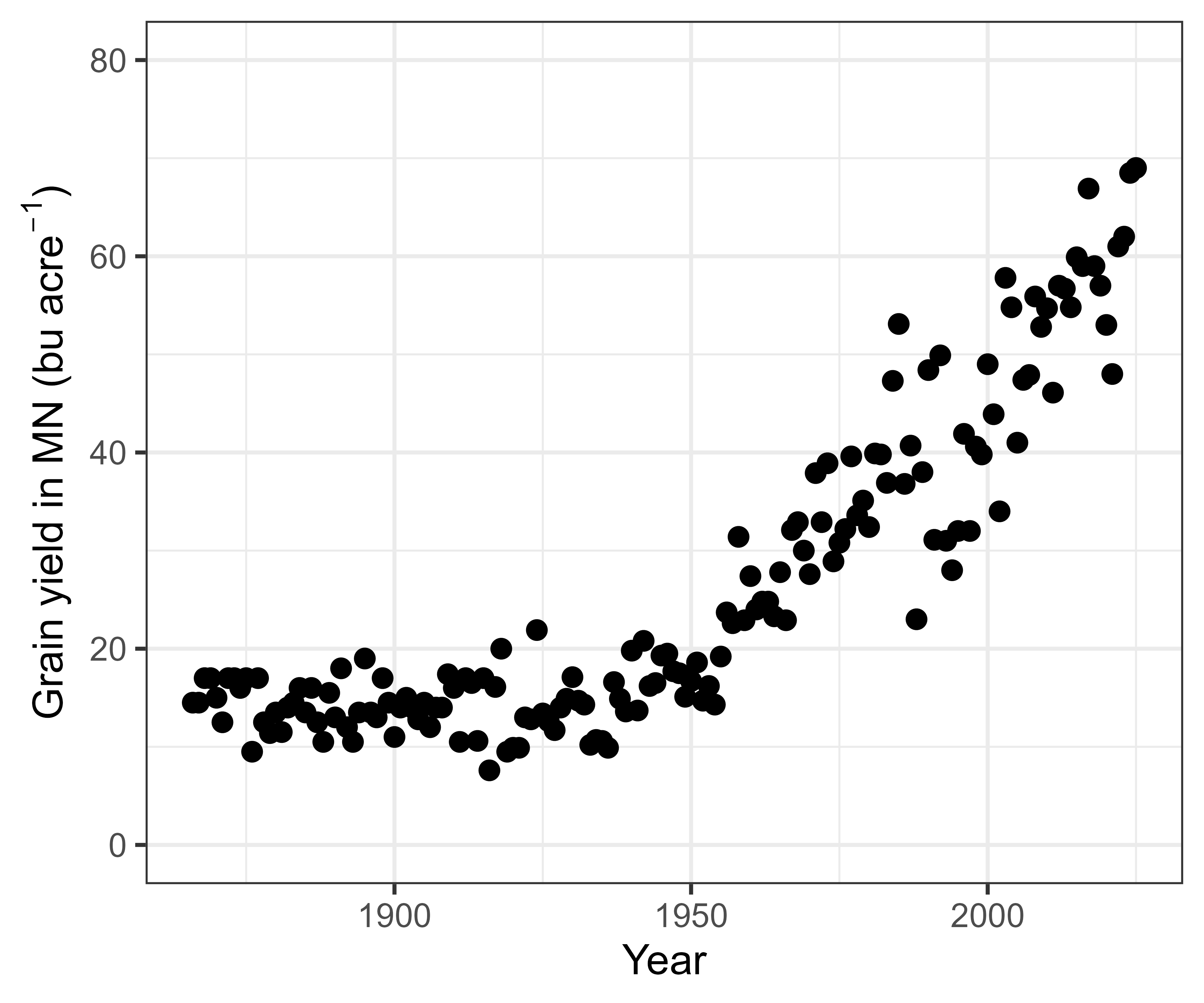


**Fig. S1.** Wheat grain yield in Minnesota (MN) between 1866 and 2025 (United States Department of Agriculture (USDA), National Agricultural Statistics Service (NASS) 2026).


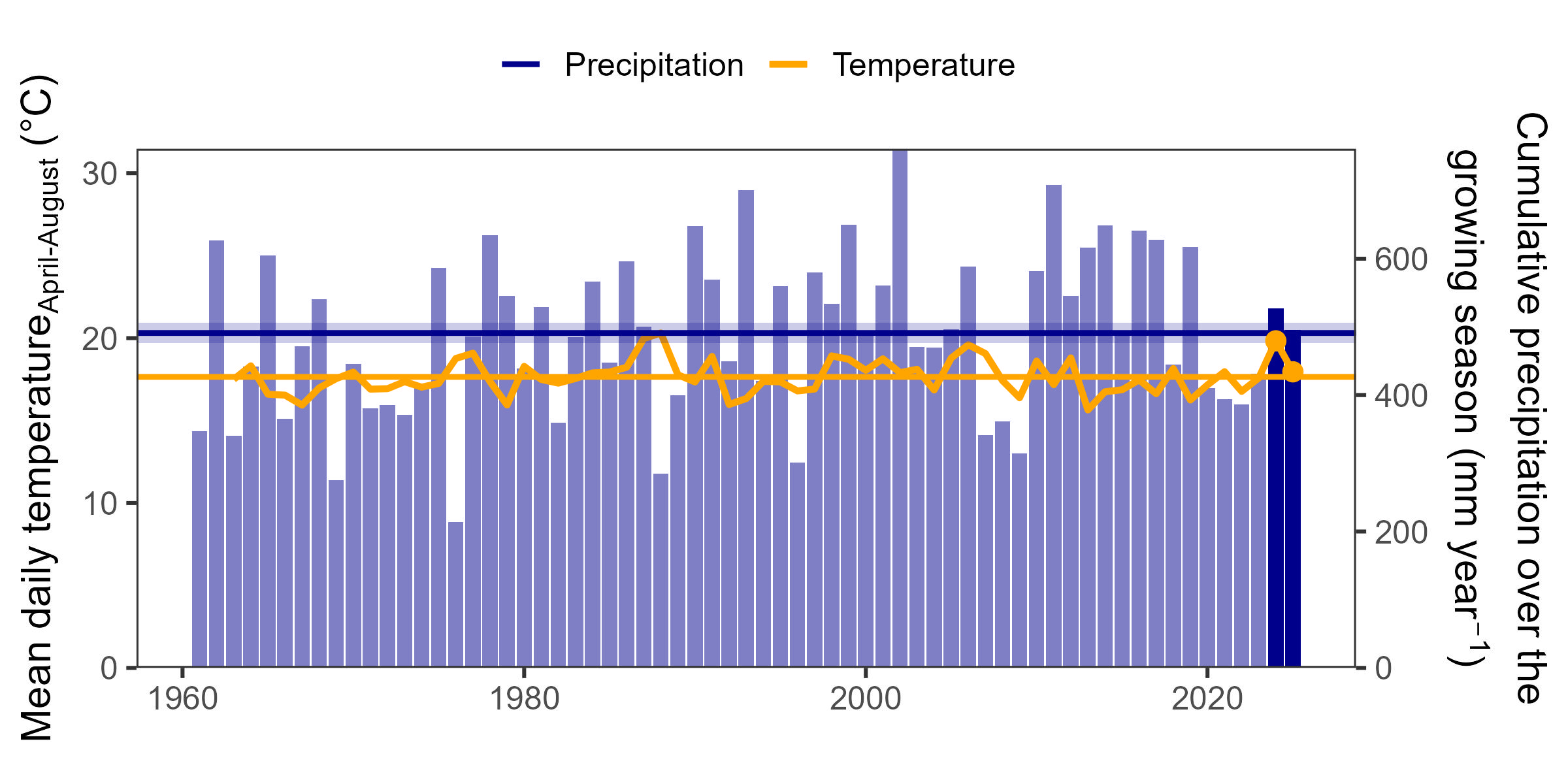


**Fig. S2.** Growing-season (April–August) mean daily temperature (orange line) and cumulative precipitation (blue bars) from 1961–2025, compared with field experiment conditions in 2024 and 2025. Horizontal lines indicate long-term mean temperature and precipitation. Orange points and dark blue bars highlight conditions during the experimental periods (2024: 14 May–19 August, dotted lines; 2025: 17 April–6 August). Data source: Department of Natural Resources, Station ID 218450 (University of Minnesota St. Paul; https://www.dnr.state.mn.us/climate/historical/daily-data.html?sid=218450&sname=UNIVERSITY%20OF%20MN%20ST.%20PAUL&sdate=por&edate=por, accessed 26 May 2026).


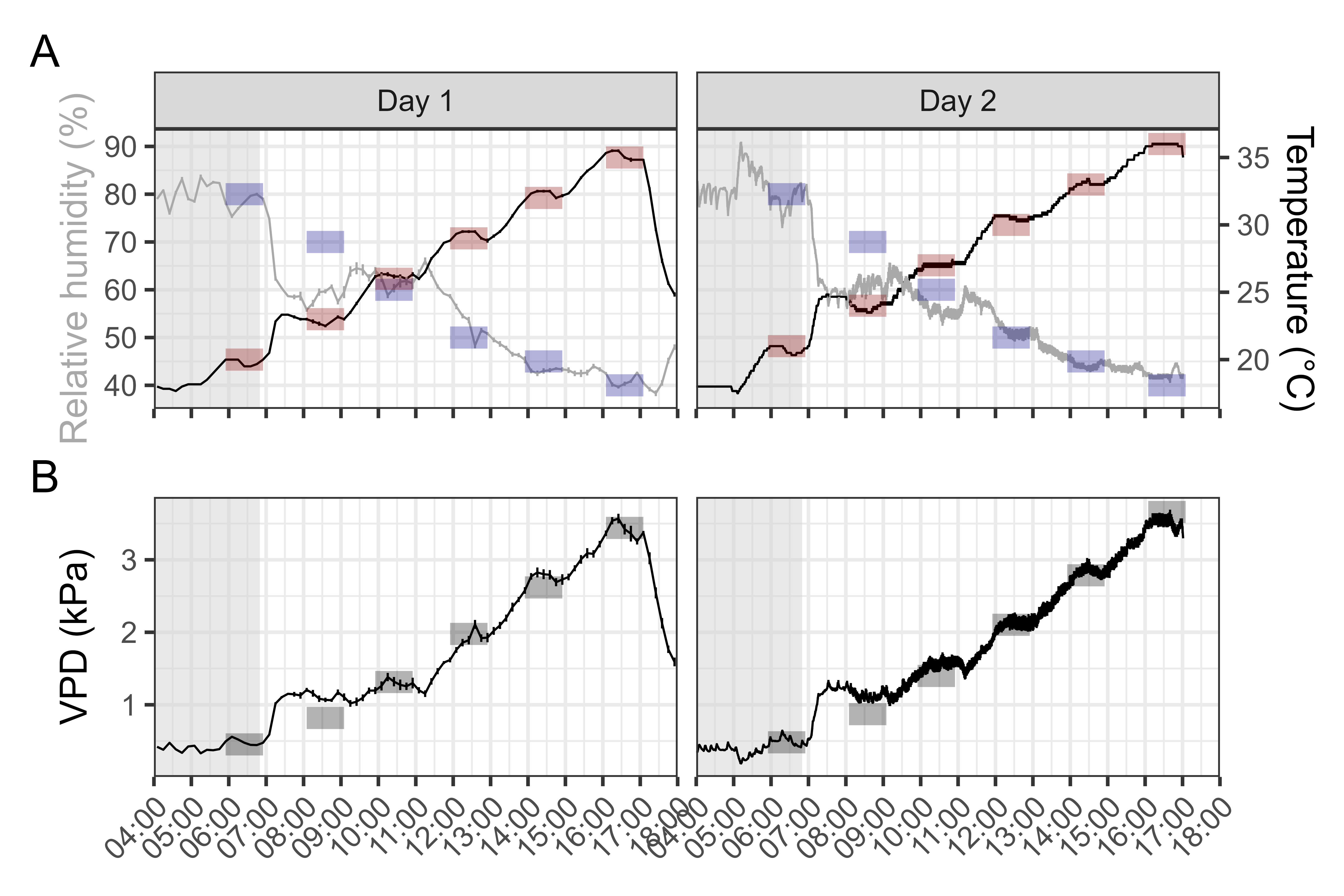


**Fig. S3.** Time course of (A) relative humidity, temperature, and (B) VPD over the experiment. The light grey shaded area marks the time before the lights were turned on. The dark grey, blue, and red shaded areas indicate the targeted VPD, relative humidity, and temperature levels, along with their respective durations.

**

Fig. S4.** Relation between stomatal density and investigated water use, hydraulic, and developmental traits: (A) the initial slope of the TR-VPD response (Slope1), (B) the VPD upon which the slope in TR changed with rising VPD (VPD_BP_), (C) the slope after the VPD_BP_ (Slope2), (D) the percentage difference in slope before and after the VPD_BP_ (Slope_diff._), (E) the total daily cumulated transpiration rate (TR_tot_), (F) the total water loss over the course of the day at the whole plant level (i.e., without normalizing by leaf area, TWL), (G) nighttime transpiration rate (TR_night_), (H) ratio between nighttime and daytime transpiration rate at the highest imposed VPD level (TR_night_ TR_day_^-1^), (I) transpiration efficiency (TE), (J) leaf area (LA), (K) specific leaf area (SLA), (L) leaf blade dry mass, (M) root biomass, (N) root:shoot ratio, (O) and plant hydraulic conductance (K_plant_). A dotted regression line indicates that the absence of a statistical association between measured traits and genotypes with increasing YOR (p ≥ 0.05).


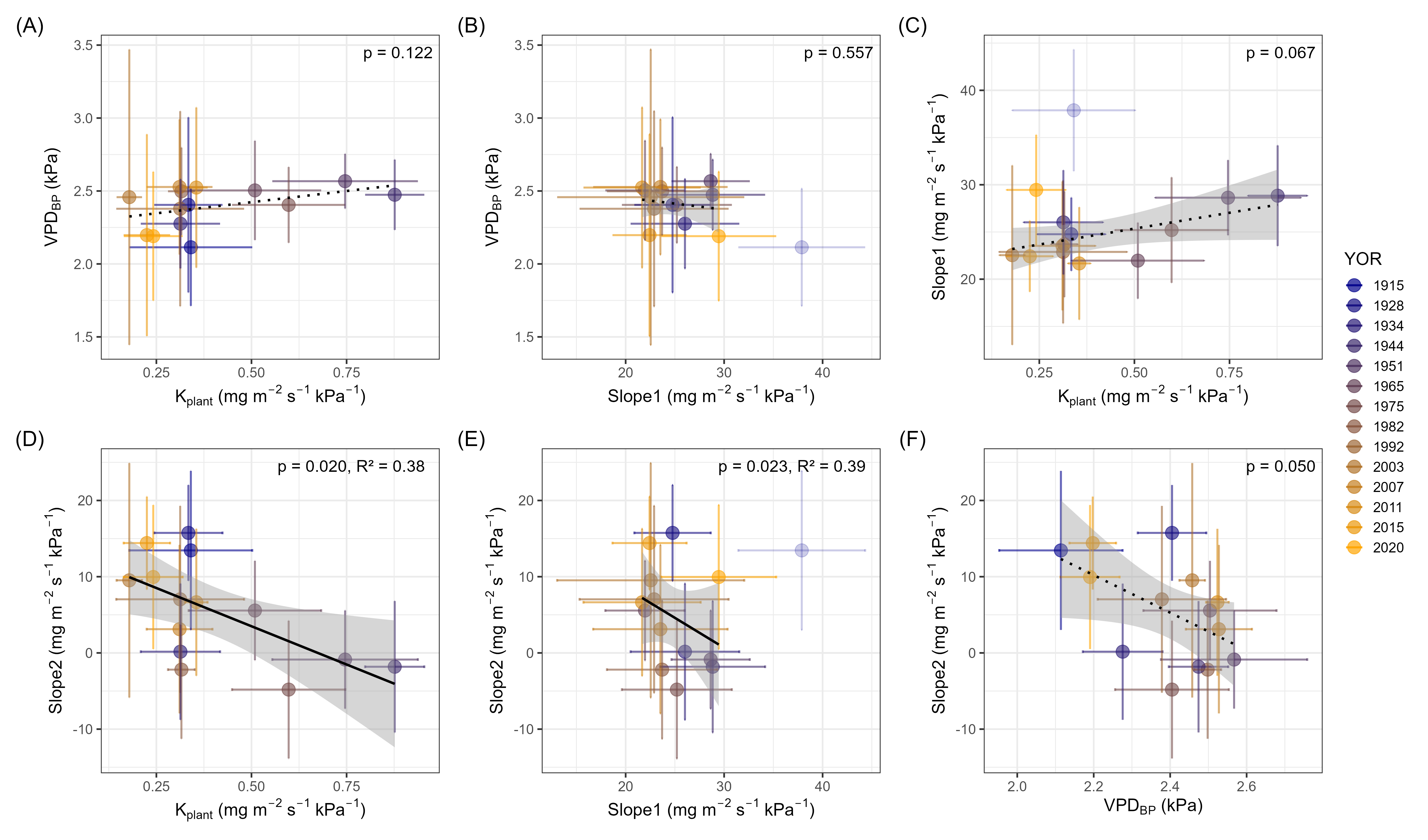


**Fig. S5.** (A) Relationship between the VPD breakpoint (VPD_BP_, i.e., the VPD at which the increase in transpiration rate becomes restricted) and the plant hydraulic conductance (K_plant_; representing hydraulic supply), and B) Slope1 (used as a proxy for maximum canopy conductance, g_c max._; representing water demand). (C) Relationship between Slope1 and K_plant_. (D) Relationship between Slope2 (i.e., the slope of the transpiration rate response to rising VPD beyond VPD_BP_) and K_plant_, and E) Slope1. (F) Relationship between two metrics of stomatal response to increasing VPD: Slope2 and VPD_BP_. Influential observations (identified based on Cook’s distance) were accounted for in all analyses. These points are displayed with increased transparency: (A) none, (B) 1915 (Glyndon), (C) 1915 (Glyndon), (D) none, (E) 1915 (Glyndon), (F) none.


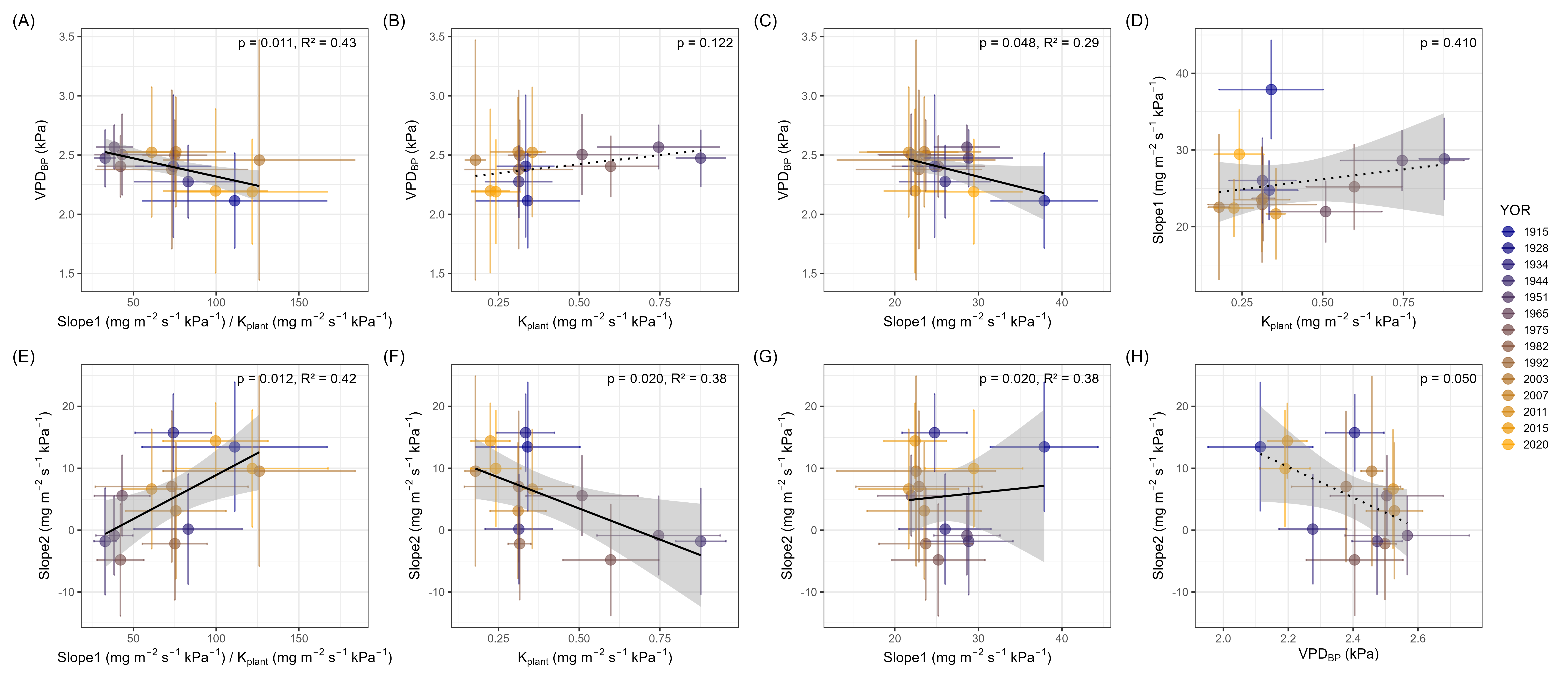


**Fig. S6.** (A) Relationship between the VPD at which the increase in the transpiration rate was restricted with rising VPD (the VPD breakpoint, VPD_BP_) and the ratio between the maximum canopy conductance (Slope1, i.e., proxy for g_c max._) and the plant hydraulic conductance (K_plant_). (B) Relationship between VPD_BP_ and K_plant_, and C) Slope1. (D) Relationship between Slope1 and K_plant_. (E) Relationship between the slope after the VPD_BP_ (Slope2) and the ratio between Slope1 and K_plant_. (F) Relationship between Slope2 and K_plant_, and G) Slope1. (H) Relationship between two metrics of stomatal response to increasing VPD: Slope2 and VPD_BP_. Influential observations (identified based on Cook’s distance) were not accounted for in the analyses.

**Table S1.** Overview of the spring wheat genotypes used in this study, their year of release (YOR) and references.

| **Cultivars** | **Year of release** | **Used in controlled-environment trial?** | **References** |
| --- | --- | --- | --- |
| Glyndon | 1915 | Yes |  |
| Reliance | 1926 | No |  |
| Marquillo | 1928 | Yes |  |
| Thatcher | 1934 | Yes |  |
| Newthatch | 1944 | Yes |  |
| Lee | 1951 | Yes |  |
| Crim | 1963 | No | (Ausemus, 1964) |
| Chris | 1965 | Yes | (Heiner and Johnston, 1967) |
| Polk | 1968 | No | (Heiner and McVey, 1971b) |
| Era | 1970 | No | (Heiner and McVey, 1971a) |
| Kitt | 1975 | Yes | (Heiner et al., 1976) |
| Angus | 1978 | No | (Elsayed et al., 1979) |
| Marshall | 1982 | Yes | (Busch et al., 1983) |
| Vance | 1989 | No | (Busch et al., 1990) |
| Norm | 1992 | Yes | (Busch et al., 1993) |
| BacUp | 1996 | No | (Busch et al., 1998) |
| McVey | 1999 | No | (Busch et al., 1998) |
| Knudson | 2001 | No |  |
| Oklee | 2003 | Yes | (Anderson et al., 2005) |
| Glenn | 2005 | No | (Mergoum et al., 2006) |
| RB07 | 2007 | Yes | (Anderson et al., 2009) |
| Sabin | 2009 | No | (Anderson et al., 2012) |
| Rollag | 2011 | Yes | (Anderson et al., 2015) |
| Linkert | 2013 | No | (Anderson et al., 2018a) |
| Bolles | 2015 | Yes | (Anderson et al., 2018b) |
| Shelly | 2016 | No | (Anderson et al., 2019) |
| Lang-MN | 2017 | No | (Anderson et al., 2021) |
| MN-Torgy | 2020 | Yes | (Anderson et al., 2024) |
| MN-Rothsay | 2022 | No | (Anderson et al., 2025) |

**Table S2.** PERMANOVA results on the transpiration rate response to increasing VPD. Significant factors (P(perm) ≤ 0.05) are shown in bold. Source: The factor or interaction being tested for its effect on the variation in the data. df: Degrees of freedom per factor. SS: Sum of Squares. MS: Mean Square. Pseudo-F: The F-statistic in PERMANOVA. P(perm): The permutation-based p-value. perms: The number of permutations used in the test.

| Source | df | SS | MS | Pseudo-F | P(perm) | perms |
| --- | --- | --- | --- | --- | --- | --- |
| **day** | 1 | 14761 | 14761 | 14.28 | 0.0001 | 9944 |
| **YOR** | 13 | 58671 | 4513.2 | 10.543 | 0.0001 | 9947 |
| day:YOR | 13 | 5565.1 | 428.08 | 0.41413 | 0.9789 | 9939 |

**Table S3.** Genotypic differences between the following traits: the total daily cumulated transpiration rate (TR_tot_), the total water loss over the course of the day at the whole plant level (i.e., without normalizing by leaf area, TWL), nighttime transpiration rate (TR_night_), ratio between nighttime and daytime transpiration rate at the highest imposed VPD level (TR_night_ TR_day_^-1^), transpiration efficiency (TE), leaf area (LA), specific leaf area (SLA), leaf blade dry mass, root biomass, root:shoot ratio, plant hydraulic conductance (K_plant_), and stomatal density. Significant factors (p < 0.05) are shown in bold.

| **Parameter** | mean | p | shapiro | bartlett | test |
| --- | --- | --- | --- | --- | --- |
| **TR_tot_ (mg m^-2^ day^-1^)** | **674995.96 ± 26181.39** | **0.017** | **0.071** | **0.973** | **ANOVA** |
| TWL (mg day^-1^) | 71034.52 ± 2070.50 | 0.527 | 0.017 | 0.810 | Kruskal-Wallis |
| **TR_night_ (mg min^-1^ cm^-2^)** | **5.28 ± 0.25** | **0.027** | **0.461** | **0.031** | **Kruskal-Wallis** |
| TR_night_ TR_day_^-1^ (-) | 0.10 ± 0.01 | 0.612 | 0.893 | 0.734 | ANOVA |
| TE (mg_biomass_ mg_water_^-1^) | 0.19 ± 0.01 | 0.774 | 0.897 | 0.514 | ANOVA |
| LA (cm^2^) | 1063.73 ± 27.24 | 0.247 | 0.095 | 0.572 | ANOVA |
| SLA (cm2 g^-1^) | 244.73 ± 4.08 | 0.119 | 0.161 | 0.948 | ANOVA |
| leaf biomass (g) | 4.35 ± 0.09 | 0.425 | 0.737 | 0.410 | ANOVA |
| root biomass (g) | 5.91 ± 0.35 | 0.663 | 0.396 | 0.164 | ANOVA |
| root:shoot ratio (g g^-1^) | 0.80 ± 0.04 | 0.550 | 0.889 | 0.680 | ANOVA |
| **K_plant_ (mg s^-1^ kPa^-1^)** | **0.40 ± 0.05** | **0.026** | **0.458** | **0.633** | **ANOVA** |
| **stomata density (mm^-2^)** | **82.51 ± 2.62** | **<0.001** | **0.921** | **0.723** | **ANOVA** |

References

Anderson, J. A.; Busch, R. H.; McVey, D. V.; Kolmer, J. A.; Linkert, G. L.; Wiersma, J. V. et al. (2005): Registration of ‘Oklee’ Wheat. In: *Crop Science* 45 (2), S. 784–785. DOI: 10.2135/cropsci2005.0784.

Anderson, J. A.; Linkert, G. L.; Busch, R. H.; Wiersma, J. J.; Kolmer, J. A.; Jin, Y. et al. (2009): Registration of ‘RB07’ Wheat. In: *J. plant regist.* 3 (2), S. 175–180. DOI: 10.3198/jpr2008.08.0478crc.

Anderson, J. A.; Wiersma, J. J.; Linkert, G. L.; Kolmer, J. A.; Jin, Y.; Dill-Macky, R. et al. (2012): Registration of ‘Sabin’ Wheat. In: *J. plant regist.* 6 (2), S. 174–179. DOI: 10.3198/jpr2011.06.0344crc.

Anderson, J. A.; Wiersma, J. J.; Linkert, G. L.; Reynolds, S.; Kolmer, J. A.; Jin, Y. et al. (2015): Registration of ‘Rollag’ Spring Wheat. In: *J. plant regist.* 9 (2), S. 201–207. DOI: 10.3198/jpr2014.07.0048crc.

Anderson, J. A.; Wiersma, J. J.; Linkert, G. L.; Reynolds, S. K.; Kolmer, J. A.; Jin, Y. et al. (2018a): Registration of ‘Bolles’ Hard Red Spring Wheat with High Grain Protein Concentration and Superior Baking Quality. In: *J. plant regist.* 12 (2), S. 215–221. DOI: 10.3198/jpr2017.08.0050crc.

Anderson, J. A.; Wiersma, J. J.; Linkert, G. L.; Reynolds, S. K.; Kolmer, J. A.; Jin, Y. et al. (2018b): Registration of ‘Linkert’ Spring Wheat with Good Straw Strength and Adult Plant Resistance to the Ug99 Family of Stem Rust Races. In: *J. plant regist.* 12 (2), S. 208–214. DOI: 10.3198/jpr2017.07.0046crc.

Anderson, J. A.; Wiersma, J. J.; Reynolds, S. K.; Caspers, R.; Linkert, G..L.; Kolmer, J. A. et al. (2019): Registration of ‘Shelly’ Hard Red Spring Wheat. In: *J. plant regist.* 13 (2), S. 199–206. DOI: 10.3198/jpr2018.07.0049crc.

Anderson, J. A.; Wiersma, J. J.; Reynolds, S. K.; Conley, E. J.; Caspers, R.; Linkert, G. L. et al. (2021): Registration of ‘Lang‐MN’ hard red spring wheat. In: *J. plant regist.* 15 (3), S. 479–489. DOI: 10.1002/plr2.20099.

Anderson, J. A.; Wiersma, J. J.; Reynolds, S. K.; Conley, E. J.; Stuart, N.; Caspers, R. et al. (2024): Registration of ‘MN‐Torgy’ spring wheat with moderate resistance to Fusarium head blight and adult plant resistance to Ug99 stem rust. In: *J. plant regist.* 18 (1), S. 122–133. DOI: 10.1002/plr2.20321.

Anderson, James A.; Wiersma, Jochum J.; Reynolds, S. K.; Conley, E. J.; Stuart, N.; Caspers, R. et al. (2025): Registration of ‘MN‐Rothsay’ spring wheat with high grain yield and lodging resistance. In: *J. plant regist.* 19 (1), Artikel e20400. DOI: 10.1002/plr2.20400.

Ausemus, E. R. (1964): Crim Wheat 1 (Reg. No. 435). In: *Crop Science* 4 (6), S. 668–669. DOI: 10.2135/cropsci1964.0011183X000400060049x.

Busch, R.; McVey, D.; Wiersma, J.; Warnes, D.; Wilcoxson, R.; Hareland, G. (1993): Registration of ‘Norm’ Wheat. In: *Crop Science* 33 (4), S. 880–881. DOI: 10.2135/cropsci1993.0011183X003300040062x.

Busch, R.; McVey, D.; Wiersma, J.; Warnes, D.; Wilcoxson, R.; Youngs, V. (1990): Registration of ‘Vance’ Wheat. In: *Crop Science* 30 (3), S. 749. DOI: 10.2135/cropsci1990.0011183X003000030068x.

Busch, R.; McVey, D.; Youngs, V.; Heiner, R.; Elsayed, F. (1983): Registration of Marshall Wheat 1 (Reg. No. 665). In: *Crop Science* 23 (1), S. 187. DOI: 10.2135/cropsci1983.0011183X002300010074x.

Busch, R. H.; McVey, D. V.; Linkert, G. L.; Wiersma, J. V.; Warnes, D. D.; Wilcoxson, R. D. et al. (1998): Registration of ‘BacUp’ Wheat. In: *Crop Science* 38 (2), S. 550–551. DOI: 10.2135/cropsci1998.0011183X003800020073x.

Elsayed, F. A.; Heiner, R. E.; McVery, D. V.; Wilcoxson, R. D. (1979): Registration of Angus Wheat 1 (Reg. No. 623). In: *Crop Science* 19 (5), S. 749–750. DOI: 10.2135/cropsci1979.0011183X001900050071x.

Heiner, R. E.; Johnston, D. R. (1967): Registration of Chris Wheat 1 (Reg. No. 462). In: *Crop Science* 7 (2), S. 170. DOI: 10.2135/cropsci1967.0011183X000700020039x.

Heiner, R. E.; McVey, D. V. (1971a): Registration of Era Wheat 1 (Reg. No. 493). In: *Crop Science* 11 (4), S. 604. DOI: 10.2135/cropsci1971.0011183X001100040067x.

Heiner, R. E.; McVey, D. V. (1971b): Registration of Polk Wheat 1 (Reg. No. 491). In: *Crop Science* 11 (4), S. 604. DOI: 10.2135/cropsci1971.0011183X001100040065x.

Heiner, R. E.; McVey, D. V.; Elsayed, F. A. (1976): Registration of Kitt Wheat 1 (Reg. No. 576). In: *Crop Science* 16 (5), S. 744. DOI: 10.2135/cropsci1976.0011183X001600050058x.

Mergoum, M.; Frohberg, R. C.; Stack, R. W.; Olson, T.; Friesen, T. L.; Rasmussen, J. B. (2006): Registration of ‘Glenn’ Wheat. In: *Crop Science* 46 (1), S. 473–474. DOI: 10.2135/cropsci2005.0287.

United States Department of Agriculture (USDA), National Agricultural Statistics Service (NASS) (2026): Quick Stats. Online verfügbar unter https://quickstats.nass.usda.gov/results/8E0E0E62-9A73-3E3F-AA29-B8DDFE94D628, accessed 22.01.2026.
